# MRSA nasal screening predictive values assessment in patients with osteomyelitis

**DOI:** 10.1017/ash.2024.368

**Published:** 2024-09-20

**Authors:** Stefanie Stramel, Valerie Buckley, MaiCuc Tran, Todd Price

**Affiliations:** 1 Pharmacy Department, Memorial Hermann Memorial City Medical Center, Houston, TX, USA; 2 Pharmacy Department, St. Luke’s Health - The Woodlands Hospital, The Woodlands, TX, USA; 3 Infectious Diseases, Memorial Hermann Memorial City Medical Center, Houston, TX, USA

## Abstract

**Objective and Design:**

The purpose of this study was to evaluate the diagnostic value of methicillin-resistant *Staphylococcus aureus* (MRSA) nasal screening utilizing polymerase chain reaction (PCR) assays in patients with osteomyelitis.

**Setting:**

A multisite, retrospective adult chart review from March 2021 to June 2022 was conducted, with no interventions performed. Patients treated with anti-MRSA therapy for osteomyelitis, MRSA nares PCR collected within 48 hours of antibiotic initiation, and related cultures were evaluated.

**Patients:**

Adults with associated cultures were assessed for concordance with MRSA nares PCR screening results. The primary outcome was the diagnostic value of MRSA nares PCR assay screening for predicting MRSA osteomyelitis. An assessment of sensitivity, specificity, positive predictive value (PPV), and negative predictive value (NPV) was performed. Key secondary outcomes included length of hospital stay and duration of anti-MRSA therapy.

**Results:**

One hundred seven patients with MRSA nares PCR sensitivities were included in this assessment. The PPV and NPV were determined to be 25% CI (10.08–49.79%) and 94.7% CI (91.29–96.87%), respectively. Median and interquartile range durations of anti-MRSA therapy were decreased in the MRSA PCR negative group at 5 (3–8) versus 6.5 (6–9.75) days (*p* = 0.03752).

**Conclusion:**

This research showed a high NPV (94.7%) and a low PPV (25%) that aligned with other studies evaluating MRSA nares PCR utilization in osteomyelitis. Additionally, despite no active intervention on the results, early de-escalation of inappropriate antibiotic therapy was observed.

## Background

Knowledge of methicillin-resistant *Staphylococcus aureus* (MRSA) colonization is a key factor in determining patient risk of infection by MRSA and subsequently aids in therapeutic decisions. Growing literature has supported the use of the MRSA nasal polymerase chain reaction (PCR) test to identify colonized individuals who may prompt a need for empiric MRSA treatment. Currently, literature is focused on the treatment of pneumonia or skin and skin structure infections showing a negative predictive value (NPV) between 80 and 97%.^1–4^ Noeldner et al recently added to that repository of knowledge with their publication regarding the use of the MRSA nasal PCR in infections beyond the respiratory tract. They specifically examined predictive values for cultures from blood, bone, and soft tissue infections.^5^ Although this literature is beneficial in adding to the increased utility of the MRSA nasal PCR, more evidence is needed in those patients presenting with osteomyelitis as <8.5% of their patients studied had bone infections.^5^

This study sought to examine the predictive values of the MRSA nasal PCR in those patients presenting specifically with osteomyelitis. Secondary outcomes assessed included length of hospital stay, duration of anti-MRSA therapy, incidence of Acute Kidney Injury (AKI) with vancomycin use, and frequency of de-escalation of anti-MRSA antimicrobial therapy.^6^

## Methods

This was a multicenter retrospective study performed across 5 hospitals in a large healthcare system in Houston, Texas. Hospital types included in this study were 1 academic medical center and 4 community hospitals of varying sizes. This study was conducted from March 1, 2021 to June 30, 2022. Patients were included if they were 18 years or older, received anti-MRSA therapy within 72 hours of hospital presentation, and had a diagnosis of osteomyelitis confirmed via associated imaging according to the Infectious Diseases Society of America (IDSA) guidance and diagnostic criteria seen in clinical practice.^8–10^ Patients also had to have the MRSA nares PCR collected within the first 48 hours of administration of anti-MRSA therapy with cultures collected in the same window. Those patients excluded were those who received anti-MRSA therapy, including mupirocin, for >48 hours prior to collection of the MRSA PCR, those with MRSA identified in any culture in the previous 30 days, those with prior hospitalization within the past 90 days, those with prior anti-MRSA therapy within the past 90 days, patients in which a MRSA nares culture was ordered instead of a swab, and patients who had culture documentation outside of our health system.

Anti-MRSA therapy was defined as any of the following antibiotics: vancomycin, linezolid, daptomycin, clindamycin, doxycycline, ceftaroline, and trimethoprim/sulfamethoxazole. Local antibiogram susceptibilities for non-urinary pathogens demonstrated methicillin resistance of ∼40% for *Staphylococcus aureus* during the course of this study. Additionally, only one encounter (the first encounter) per patient was included for analysis.

AKI was defined according to Kidney Disease Improving Global Outcomes (KDIGO) guideline.^6^

### Statistical analysis

Data were analyzed with descriptive statistics using medians and proportions. Sensitivity, specificity, positive predictive value (PPV), and NPV of MRSA nasal PCR were also analyzed for estimation. χ^2^ test was utilized to assess differences in growth of MRSA in culture and the MRSA nares PCR.

## Results

Data analysis included 107 patients with MRSA nares PCR results, associated cultures, and imaging confirming osteomyelitis with no other excluding factors present. Median and interquartile range (IQR) age in years was 58 (49–67), and the population was predominantly male at 80.4%. Patients were identified with osteomyelitis via magnetic resonance imaging (MRI) at 78.5% followed by radiograph at 15% and computed tomography (CT) at 6.5% and most frequently had osteomyelitis of the foot at 71% followed distantly by the toe at 12.1%. Complete patient characteristics are in Table 1.

**Table 1.** Patient characteristics

Characteristic	Positive PCR	Negative PCR	
**Number of patients, no. (%)**	12 (11.2)	95 (88.8)	–
**Age (yrs.), median (IQR)**	61.5 (55.3–70)	58 (49–66)	*p* = 0.200
**Male, no. (%)**	11 (91.7)	75 (78.9)	*p* = 0.296
**Imaging type, no. (%)**			
Radiograph	0	16 (16.8)	
MRI	12 (100)	72 (75.8)	
CT	0	7 (7.4)	
**Osteomyelitis, no. (%)**			
Foot	10 (83.3)	69 (72.6)	
Knee	0	1 (1.1)	
Other	2 (16.7)	25 (26.3)	
**No. patients from each hospital, no. (%)**			
Academic Medical Center	0	2 (2.1)	
Large Community	9 (75)	52 (54.8)	
Small Community	3 (25)	41 (43.1)	
**Culture results, no. (%)**			
MRSA	3 (25)	4 (4.2)	
MSSA	3 (25)	23 (24.2)	
*Staphylococcus* spp.	1 (8.3)	0	
Gram-negative	2 (16.7)	9 (9.5)	
Polymicrobial	2 (16.7)	35 (36.8)	
Negative	1 (8.3)	18 (18.9)	
Other	0	6 (6.4)	
**Therapy by site, no. (%)**			
Foot			
Vancomycin	9 (75)	65 (68.4)	
Clindamycin	1 (8.3)	4 (4.2)	
Knee			
Vancomycin	0	1 (1.1)	
Clindamycin	0	0	
Other			
Vancomycin	1 (8.3)	23 (24.1)	
Clindamycin	1 (8.3)	1 (1.1)	
Linezolid	0	1 (1.1)	

MRSA nares PCR sensitivities were found to show a determination of 37.5% CI (8.52–75.51%) alignment with cultures. A specificity of 90.9% CI (83.44–95.76%), a PPV of 25% CI (10.08–49.79%), and a NPV of 94.7% CI (91.29–96.87%) are described in Table 2. Included secondary outcomes in Table 3 showed median (IQR) length of hospital stay of 7.07 (4.36–10.23) versus 8.24 (6.91–12.2) (*p* = 0.17068), median (IQR) durations of anti-MRSA therapy were decreased in the MRSA PCR negative group at 5 (3–8) versus 6.5 (6–9.75) days (*p* = 0.03752). Patients with an incidence of AKI with anti-MRSA therapy showed 24 patients in those with a negative MRSA nares PCR versus 6 in those who had a positive MRSA nares PCR resulted (*p* = 0.072). These findings can be seen in Table 3. Frequency of de-escalation of anti-MRSA antimicrobial therapy was not found to be statistically significant at *p* = 0.186 among groups.

**Table 2.** Primary outcomes

Statistic	Value	95% CI
**Negative predictive value**	94.74%	91.29 to 96.87%
**Positive predictive value**	25.00%	10.08 to 49.79%
**Sensitivity**	37.50%	8.52 to 75.51%
**Specificity**	90.91%	83.44 to 95.76%

**Table 3.** Secondary outcomes

Outcome	Positive PCR	Negative PCR	
**Length of stay, days, median (IQR)**	8.24 (6.9–12.2)	7.07 (4.4–10.2)	*p* = 0.171
**Duration of therapy, days, median (IQR)**	6.5 (6–9.8)	5 (3–8)	*p* = 0.03
**De-escalation**	2	34	*p* = 0.186
**Incidence of AKI**	6	24	*p* = 0.072

## Discussion

MRSA nasal PCR in osteomyelitis showed high specificity of 90.1% and high NPV of 94.7%. These findings were similar to other studies, albeit the others are not directly related to osteomyelitis. These data further contribute to the growing evidence that MRSA nasal PCR assays are a useful tool in ruling out MRSA infections in those patients presenting with osteomyelitis. Of note, the sensitivity was lower at 37.5% compared to 55.0% (31.5–76.9) found by Noeldner et al.^5^ No statistical difference was seen with respect to length of stay, or those developing an AKI. These findings are presumed to be due to no interventions made upon finalization of results. However, a difference was seen in median durations of anti-MRSA therapy despite no intervention on MRSA PCR results being performed. It is theorized that, with the incorporation of education and protocols, future observations may be seen.

### Limitations

During this period, a quality improvement project was initiated at the primary research location promoting providers to opt in to prospective MRSA nasal PCR orders for patients with known or suspected osteomyelitis. This project prompted this retrospective analysis. This test is currently not part of any order set and was included as a quality improvement project outcome with orders from select physicians being actively placed at only 1 of the included community facilities. However, several physicians practice and/or collaborate with other physicians at other included hospitals which may have led to the ability to have findings at our other facilities. The initiative relied on most orders being placed, without real-time final result review, by the Infectious Diseases Clinical Pharmacy Specialist during normal business hours. This constraint may have affected time-sensitive secondary outcomes. Furthermore, the microbiology lab is located at two centralized locations and specimen transport times may have also affected time-sensitive secondary outcomes. Education was also still being disseminated to providers regarding low PPVs for MRSA PCR nares in reference to other infection types. Therefore, not all providers may be familiar or trust the clinical applicability of the results leading to inconsistent de-escalation.

## Conclusion

The MRSA PCR nares high NPV of 94.7% in patients with osteomyelitis has. This study also saw a decrease in anti-MRSA therapy durations, despite no recommended discontinuation interventions. Future studies with active discontinuation interventions being performed may affect the included secondary outcomes herein, as one hospital is now performing per protocol.
